# Bluues2: An enhanced web server for fast and accurate electrostatic analysis of proteins and protein complexes

**DOI:** 10.1002/pro.70815

**Published:** 2026-10-01

**Authors:** Damiano Piovesan, Nima Rasekh, Miguel Angel Soler, Walter Rocchia, Gennaro Esposito, Federico Fogolari, Silvio C. E. Tosatto

**Affiliations:** ^1^ Department of Biomedical Sciences University of Padua Padua Italy; ^2^ Department of Molecular Sciences and Nanosystems Ca' Foscari University of Venice Venice Italy; ^3^ CONCEPT Lab Istituto Italiano di Tecnologia Genoa Italy; ^4^ Istituto Nazionale Biostrutture e Biosistemi Rome Italy; ^5^ Science and Math Division New York University at Abu Dhabi Abu Dhabi United Arab Emirates; ^6^ Department of Mathematics, Computer Science and Physics University of Udine Udine Italy

**Keywords:** generalized born, solvent models, web server

## Abstract

Electrostatic interactions play a crucial role in protein structure, stability, and function. We present Bluues2, a significantly enhanced web server for calculating electrostatic properties of proteins using the Generalized Born model. The new version features improved accuracy through refined GB radius calculations using NanoShaper for molecular surface generation, extended functionality for analyzing protein‐protein and protein‐ligand complexes including calculation of binding free energies and electrostatic complementarity, enhanced interface with integrated Mol* structure viewer, and compatibility with the DRMAAtic framework for efficient job management. Bluues2 provides comprehensive electrostatic analyses including pKa predictions, surface potentials, solvation free energies, and complex interface properties in a user‐friendly format accessible to both experts and non‐specialists.

## 1 INTRODUCTION

Electrostatic interactions are fundamental to virtually all biological processes involving proteins, influencing folding pathways, conformational stability, molecular recognition, enzymatic catalysis, and signal transduction (Alexov et al., 2011; Warshel et al., 2006). The charged and polar nature of amino acid side chains, combined with the heterogeneous dielectric environment created by the protein interior and surrounding aqueous solution, generates complex electrostatic fields that can extend over significant distances. Understanding and accurately calculating these electrostatic properties is therefore essential not only for comprehending protein function at a molecular level but also for rational protein design, enzyme engineering, and structure‐based drug discovery (Fersht et al., 1985; Strickler et al., 2006).

The Poisson‐Boltzmann (PB) equation provides a rigorous continuum model that describes the electrostatic potential around a solute immersed in an ionic solution, treating the protein as a low‐dielectric medium and water as a high‐dielectric medium with dissolved ions. Despite its widespread application and demonstrated utility, solving the Poisson‐Boltzmann equation numerically requires significant computational resources, particularly for large macromolecular assemblies or when extensive conformational sampling is required. The calculation involves discretizing the system onto a grid and solving the resulting finite‐difference equations, a process that becomes increasingly expensive as system size grows or when high accuracy is demanded through fine grid spacing.

Generalized Born (GB) models emerged as an attractive alternative to full Poisson‐Boltzmann solutions, offering a fast analytical approximation that captures the essential physics of electrostatic solvation (Bashford & Case, 2000; Chen et al., 2008; Onufriev & Case, 2019). The GB approach estimates the electrostatic solvation free energy by representing each atom with an effective Born radius that characterizes its degree of burial within the low‐dielectric protein interior. These Born radii are then used to calculate both self‐solvation energies and pairwise interaction energies in a way that approximately accounts for the screening effect of the high‐dielectric solvent. The many‐body nature of interactions is hidden in the GB radii. The computational efficiency of GB models, typically orders of magnitude faster than numerical Poisson‐Boltzmann solutions, has made them the method of choice for implicit solvent molecular dynamics simulations and has enabled extensive conformational sampling that would be impractical with more rigorous approaches.

We previously developed Bluues (Fogolari et al., 2012), a program implementing the GBR6 surface integral approach for computing electrostatic properties of proteins, based on the work of Grycuk (Grycuk, 2003) and validated by Mongan and colleagues (Mongan et al., 2007). The initial command‐line version demonstrated good agreement with Poisson‐Boltzmann calculations while maintaining computational efficiency, completing analyses in minutes rather than hours. We subsequently released a web server version that made these capabilities accessible to users without computational expertise, featuring calculation of pKa values, surface potentials, and pH‐dependent properties (Walsh et al., 2012).

Here, we present Bluues2, a substantially redesigned and enhanced version that addresses limitations of the original implementation while adding major new capabilities. The improvements span multiple dimensions. First, we have enhanced the accuracy of the underlying calculations by integrating NanoShaper (Decherchi & Rocchia, 2013), a state‐of‐the‐art tool for molecular surface generation that provides more precise and robust triangulation of the solvent‐excluded surface, particularly important for handling internal cavities and complex surface geometries. Second, we have extended the scope of analyses beyond single proteins to encompass molecular complexes, implementing a comprehensive framework for evaluating electrostatic contributions to binding in protein‐protein and protein‐ligand systems (Soler et al., 2025). This includes calculation of binding free energy components, identification of interfacial contacts, and quantification of electrostatic complementarity through correlation analysis of surface potentials at interacting interfaces.

Third, we have modernized the web infrastructure using current technologies including the Angular framework (https://angular.dev/) for the frontend interface, REST APIs for client‐server communication, and the DRMAAtic system (del Conte et al., 2025) for robust job management and scheduling. The integration of the Mol* molecular viewer (Sehnal et al., 2021) provides users with interactive three‐dimensional visualization of structures and calculated properties directly within the browser. Fourth, we have improved the organization and presentation of results to facilitate interpretation by both expert and novice users, with tabular summaries, sortable lists of residues by various criteria, and downloadable files in standard formats for further analysis.

The extension to protein complexes addresses a particularly important gap in available tools. While several servers exist for analyzing electrostatics of individual proteins, few provide integrated analysis of intermolecular electrostatic interactions. Understanding how electrostatics contributes to molecular recognition and binding is crucial for many applications, from rationalizing mutagenesis experiments to guiding the design of protein therapeutics or peptidomimetic drugs. The ability to quantify electrostatic complementarity at interfaces, identify specific residues contributing to binding through electrostatic interactions, and decompose binding free energies into interpretable components provides insights that complement structural information and binding affinity measurements.

### 1.1 Related work

Several tools already address aspects of macromolecular electrostatics, but they differ from Bluues2 in the physical model used, the type of output returned, and their support for complexes. The most widely used electrostatics packages, including APBS and DelPhi, solve the Poisson‐Boltzmann equation numerically (Jurrus et al., 2018; Rocchia et al., 2001; Zhang et al., 2025). These approaches provide a detailed continuum‐electrostatics reference, but they are computationally more demanding than GB approximations and do not naturally expose atom‐specific Born radii or pairwise decompositions. Bluues2 instead uses a GBR6 Generalized Born formulation, retaining good agreement with Poisson‐Boltzmann benchmarks while providing fast calculations and interpretable atom‐, residue‐, and surface‐level quantities.

Other web servers use GB/SA or MM/GBSA frameworks for complex analysis. HawkDock 2 combines docking with MM/GBSA‐based scoring and mutation analysis, including solvation terms based on the OBC model (Onufriev et al., 2004; Zhang et al., 2025). Its focus is on generating or ranking docking poses and estimating mutation effects. Bluues2 is complementary: it analyzes a user‐provided single structure or complex and returns a detailed electrostatic characterization of the system, including Born radii, solvation terms, pKa‐related quantities, surface potentials, interface contacts, and downloadable point‐ and atom‐level data.

GBNSR6 is the closest methodologically related program, because it also implements an R6 surface‐integral Generalized Born approach for solvation and binding‐energy calculations (Forouzesh et al., 2017). The main differences are practical and methodological. GBNSR6 approximates the surface integral with a Cartesian grid‐based representation and uses an ALPB expression for the solvation free energy, whereas Bluues2 builds triangulated molecular surfaces with NanoShaper, or optionally MSMS, and uses the GBR6 formulation implemented in Bluues. In addition, Bluues2 exposes these calculations through a web interface and extends them with pKa, surface‐potential, contact, and complex‐interface analyses.

Electrostatic complementarity at protein interfaces has also been studied with alternative surface descriptors. For example, Grassmann et al. described electrostatic and shape complementarity using two‐dimensional Zernike polynomial expansions of projected surface patches (Grassmann et al., 2023). Bluues2 follows a different strategy: it directly pairs contacting surface points at the interface and reports the Pearson correlation between their electrostatic potentials, enabling straightforward interpretation of favorable positive‐negative matching between partners. PEP‐Patch provides another related view by identifying contiguous positive or negative electrostatic‐potential patches on protein surfaces, which is useful for recognizing potential complementary regions (Hoerschinger et al., 2023).

In summary, Bluues2 does not aim to replace Poisson‐Boltzmann solvers, docking servers, or specialized surface‐patch tools. Its contribution is to combine a fast and accurate GBR6‐based electrostatics engine with a single web interface for both single proteins and complexes, delivering detailed atom‐, residue‐, surface‐, and interface‐level results that are otherwise scattered across several programs.

## 2 METHODS

Bluues2 implements the Generalized Born model for calculating electrostatic properties, using the surface integral formulation for Born radii calculation. The theoretical framework and computational methodology have been described in detail in our previous publications (Fogolari et al., 2012; Soler et al., 2025).

The reader is referred to the latter works for theoretical details and for validation compared to the reference Poisson‐Boltzmann calculations. Briefly, the GB model approximates the electrostatic solvation free energy by assigning each atom an effective Born radius that depends on its position within the molecular structure. These radii are computed using the GBR6 surface integral approach, which has been shown to provide excellent agreement with Poisson‐Boltzmann calculations for both self‐energies and pairwise interaction energies (Mongan et al., 2007; Tjong & Zhou, 2007). The method treats the protein interior as a low‐dielectric medium and the surrounding aqueous solution as a high‐dielectric medium, with ionic strength effects incorporated through modified screening functions.

### 2.1 Core electrostatic calculations

For single protein structures, Bluues2 and Bluues_cplx call Nanoshaper to generate the molecular surface, as detailed in the next section. The resolution used for surface calculation is 20 vertex points per Å^2^. The latter value was found to guarantee convergence in the computed energies of binding. The cutoff to list neighbors is 20 Å. The energy of binding computed by Bluues_cplx amounting to tens of kJ/mol is obtained by difference of very large (thousands kJ/mol) so that, in general, changing the cutoff has effects also on the computed free energy differences.

The surface of all atoms within the cutoff is used to calculate Born radii via surface integral. Using Born radii for all atoms, electrostatic solvation free energies are computed using the formula by Still et al. (Still et al., 2002). Calculations of pH‐dependent properties, including pKa values of ionizable groups, employ a Monte Carlo approach to sample protonation states at different pH values, properly accounting for coupling between ionizable sites and using an elevated interior dielectric constant (Forouzesh et al., 2017) to compensate for the neglect of conformational flexibility in the fixed‐structure model, following an established protocol described by Antosiewicz et al. (Antosiewicz et al., 1994). The surface potential calculation uses a test charge with a radius (0.7 Å) optimized to reproduce Poisson‐Boltzmann potentials (Fogolari et al., 2012).

### 2.2 Molecular surface generation

A key enhancement in Bluues2 is the integration of NanoShaper for molecular surface generation. NanoShaper uses a sophisticated ray‐casting algorithm specifically designed for biomolecular applications, providing robust handling of internal cavities and high‐quality triangulated surfaces. The program builds an alpha‐shapes‐based mathematical model of the solvent‐excluded surface by dividing space into Voronoi cells, building the so‐called mixed complex, and calculating surface patch equations for each cell (Decherchi & Rocchia, 2013). Rays are cast to identify surface intersections, which are used both to triangulate the surface and to assign surface areas and normal vectors needed for the Born radius calculation. The algorithm separately identifies external surface components and internal cavities, which can be retained or discarded depending on the application. For compatibility with existing workflows, Bluues2 also supports MSMS as an alternative surface generation tool.

### 2.3 Complex analysis

The bluues_cplx module analyzes protein‐protein and protein‐ligand complexes by performing three separate calculations: one for the intact complex and one for each isolated component. By comparing these calculations, various properties of the interface and binding energetics are extracted (Soler et al., 2025). The electrostatic component of the binding free energy is decomposed into interpretable contributions including direct Coulombic interactions, desolvation penalties for burying charges, and solvation effects on charge‐charge interactions. Additionally, a hydrophobic term proportional to buried solvent‐accessible surface area represents non‐electrostatic driving forces.

These binding‐energy components should be interpreted as approximate descriptors rather than absolute binding affinities. They do not include conformational entropy, explicit‐solvent rearrangements, or ion‐specific effects, and they are most informative when comparing closely related complexes analyzed with the same settings.

An important analysis performed by bluues_cplx is the characterization of electrostatic complementarity at interfaces. The program identifies surface points on each molecule that come within a defined cutoff distance in the complex and records pairs of potential values computed for the isolated molecules. The Pearson correlation coefficient between these paired potential values quantifies electrostatic complementarity. A strongly negative correlation indicates that regions of positive potential on one molecule align with regions of negative potential on the partner, suggesting electrostatically favorable binding. This surface potential complementarity metric has been shown to correlate with binding affinity and can distinguish native interfaces from incorrect docking poses (Soler et al., 2025).

The program also identifies atomic contacts at the interface, recording pairs of atoms whose van der Waals surfaces come within the cutoff distance. For each contact pair, atomic charges and distances are recorded, and statistical analysis including charge correlation coefficients provides a complementary measure of interface properties. All interface data are presented in organized tables and downloadable files, with coordinates of contacting atoms provided in PDB format with surface average potentials encoded in the temperature factor field for visualization.

The default cutoff for interfacial‐contact enumeration is a 1.0 Å gap between van der Waals surfaces; for two aliphatic carbon atoms, this corresponds to an interatomic distance of approximately 4.8 Å.

### 2.4 Web server architecture

Bluues2 employs a modern client‐server architecture with clear separation between the user‐facing frontend and the computational backend. The backend is built on the DRMAAtic framework, an in‐house solution that provides comprehensive job management functionality. DRMAAtic interfaces with the SLURM workload manager through the Distributed Resource Manager Application API, handling job submission, monitoring, and result retrieval. The framework is built on Django REST Framework, providing RESTful APIs for communication between the web interface and compute cluster.

User authentication is implemented through OAuth 2.0 using ORCID as the identity provider, allowing users to log in with existing credentials without separate password management. Authentication is required for job submission to enable usage tracking and prevent abuse, but results remain accessible through secure tokens for sharing. The system implements throttling mechanisms to limit simultaneous jobs per user and total system load, ensuring fair resource allocation.

The frontend uses the Angular framework to provide a responsive single‐page application that works across devices. Structure input can be provided as PDB identifiers (automatically fetched), uploaded PDB files, or PQR files with pre‐assigned parameters. For complexes, users specify which chains belong to each molecular entity through a simple selection interface. The integration of the Mol* molecular viewer enables interactive three‐dimensional visualization with computed properties mapped directly onto structures. Custom coloring schemes highlight residues of interest, and users can explore structures while examining numerical results. The ngx‐mol‐viewers component library provides additional capabilities for displaying sequence‐based data such as pKa shifts or surface potential profiles.

### 2.5 Input preparation and results

When PDB structures are provided, the server uses PDB2PQR to convert them to PQR format, adding hydrogen atoms, assigning atomic charges and radii from molecular mechanics forcefields, and optimizing the orientation and protonation state of certain groups based on local hydrogen bonding. For complex analysis, chain identifiers are used to assign atoms to molecular entities. Users who prefer complete control over parameters can upload PQR files directly, which is particularly useful for non‐standard residues or ligands not parameterized in standard forcefields.

For AlphaFold and other predicted models, users should inspect confidence and disorder annotations before electrostatic analysis. Long low‐confidence or intrinsically disordered regions, especially terminal tails, should be trimmed or interpreted with caution because a single arbitrary conformation can produce misleading electrostatic potentials.

Results are organized into multiple sections addressing different aspects of the analysis, with summary overviews, detailed atomic and residue‐level data, graphical visualizations, and comprehensive downloadable files. Figure 1 shows representative output from both server modes. Extensive documentation explains the scientific background, output interpretation, and provides tutorial examples for common analysis scenarios. The server is actively maintained by the BioComputingUP laboratory at the University of Padua, part of the ELIXIR European research infrastructure, with dedicated personnel and sustained funding ensuring continued availability and development.

**FIGURE 1:**
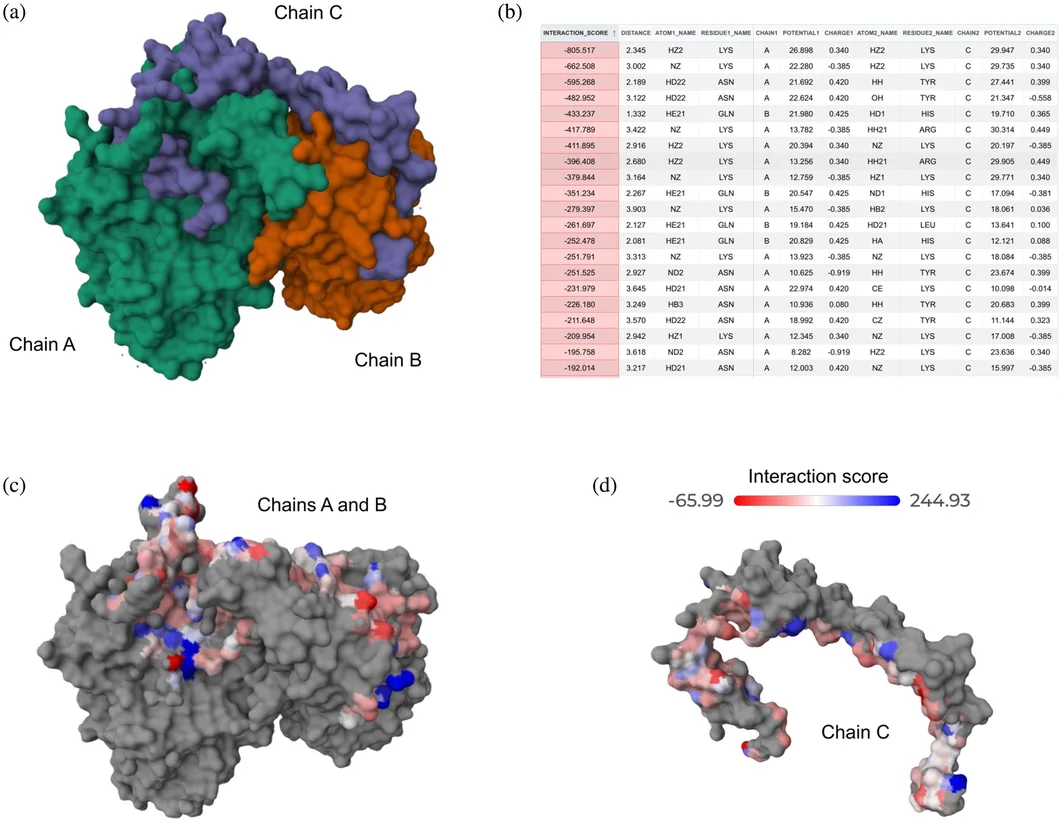
Representative Bluues2 results. (a) Structure view for the p27Kip1‐cyclin A‐Cdk2 complex (PDB ID 1JSU), colored by chain. (b) Numerical result summary and a representative subset of atom‐level output columns. (c, d) Partner‐facing interface surfaces from the complex‐mode analysis. The interaction score is shown with identical numerical limits and a shared color bar in panels c and d. Complete numerical tables and downloadable output files are available from the result page.

### 2.6 Performance and validation

Bluues2 has been systematically benchmarked against reference Poisson‐Boltzmann calculations. As reported in our previous work, the GBR6 surface integral approach provides excellent agreement with Poisson‐Boltzmann self‐energies (correlation coefficient 0.995) and solvation energies (correlation coefficient 0.9996) across a diverse set of 55 proteins spanning various sizes and structures. Surface potential calculations show good correlation (0.90) with Poisson‐Boltzmann solutions, with discrepancies mainly occurring in regions of high curvature or at boundaries where continuum models have inherent limitations.

For pKa predictions on the Gunner test set of 238 experimental values from 23 proteins, Bluues2 achieves an overall root mean square deviation of 1.00 pH units with a correlation coefficient of 0.51, comparable to other physics‐based prediction methods. Performance varies by residue type, with best results for histidine (correlation 0.74, RMSD 0.98) and typical accuracies for aspartate and glutamate residues (RMSD 0.78‐0.90). These results are consistent with the accuracy expected from single‐conformation continuum models and comparable to widely used empirical methods like PROPKA.

Because Bluues_cplx is based on differences between large electrostatic free energies, its validation is more stringent than that required for single‐structure Bluues2 calculations. Binding free energies are small differences between large absolute quantities, so even modest numerical errors can affect the final value; the same sensitivity is also encountered in Poisson‐Boltzmann calculations.

Absolute binding free energies computed by Bluues_cplx should therefore be interpreted with caution. The model does not include entropic contributions, and intra‐ and intermolecular interaction terms are treated approximately. The server is best used to analyze electrostatic potentials at interfaces and to compare trends within series of closely related complexes, where systematic errors are expected to partially cancel. Convergence was tested with respect to the surface‐point density; a density of 20 points per A^2^ was adopted because it gave stable binding‐energy trends. The use of a 2 nm cutoff did not significantly alter trends among similar complexes, although absolute values may change.

To assess whether Bluues_cplx reproduces reference Poisson‐Boltzmann calculations with sufficient accuracy, we used APBS (Jurrus et al., 2018) to compute electrostatic binding free energies on a 257 × 257 × 257 grid with a grid spacing of 0.05 nm.

The results were compared with the same quantities calculated with Bluues_cplx using matching implicit‐solvent parameters and no cutoff. Following our previous validation strategy (Soler et al., 2025), we considered 756 non‐redundant binary protein‐protein complexes from the curated PDBbind set (Wang et al., 2004). We removed 24 complexes whose dimensions exceeded the grid and 6 additional complexes with very large discrepancies between the two calculations, likely arising from differences in the molecular‐surface definitions used by the two programs. For the remaining 726 complexes, the root mean square deviation was 6.3 kJ/mol and the Pearson correlation coefficient was 0.9993 across a binding‐free‐energy range of approximately 1700 kJ/mol; see Data S1, including per‐complex energies and APBS output files for the complex and both isolated partners.

With the 2 nm cutoff used for routine server calculations, computational performance represents a key advantage of the GB approach. Typical calculations for a very large protein (PDB ID 3MK3, ~9000 residues, ~66,000 atoms) including pKa calculations complete in less than 8 min on a single CPU core. These timescales are entirely practical for interactive web use and fast enough to enable screening of multiple mutations or analysis of structural ensembles. Jobs are queued through the SLURM system with typical queue times under 1 min during normal load, and completed results remain accessible through unique URLs for 30 days.

### 2.7 Test case

To illustrate a step‐by‐step protein‐engineering workflow with Bluues_cplx, we use a retrospective test case to identify a mutation in one subunit of a complex that could destabilize binding as illustrated in Figure 2. Barnase/Barstar is suitable for this example because it has been studied extensively and many measured binding affinities are available in the SKEMPI database (Moal & Fernández‐Recio, 2012).

**FIGURE 2:**
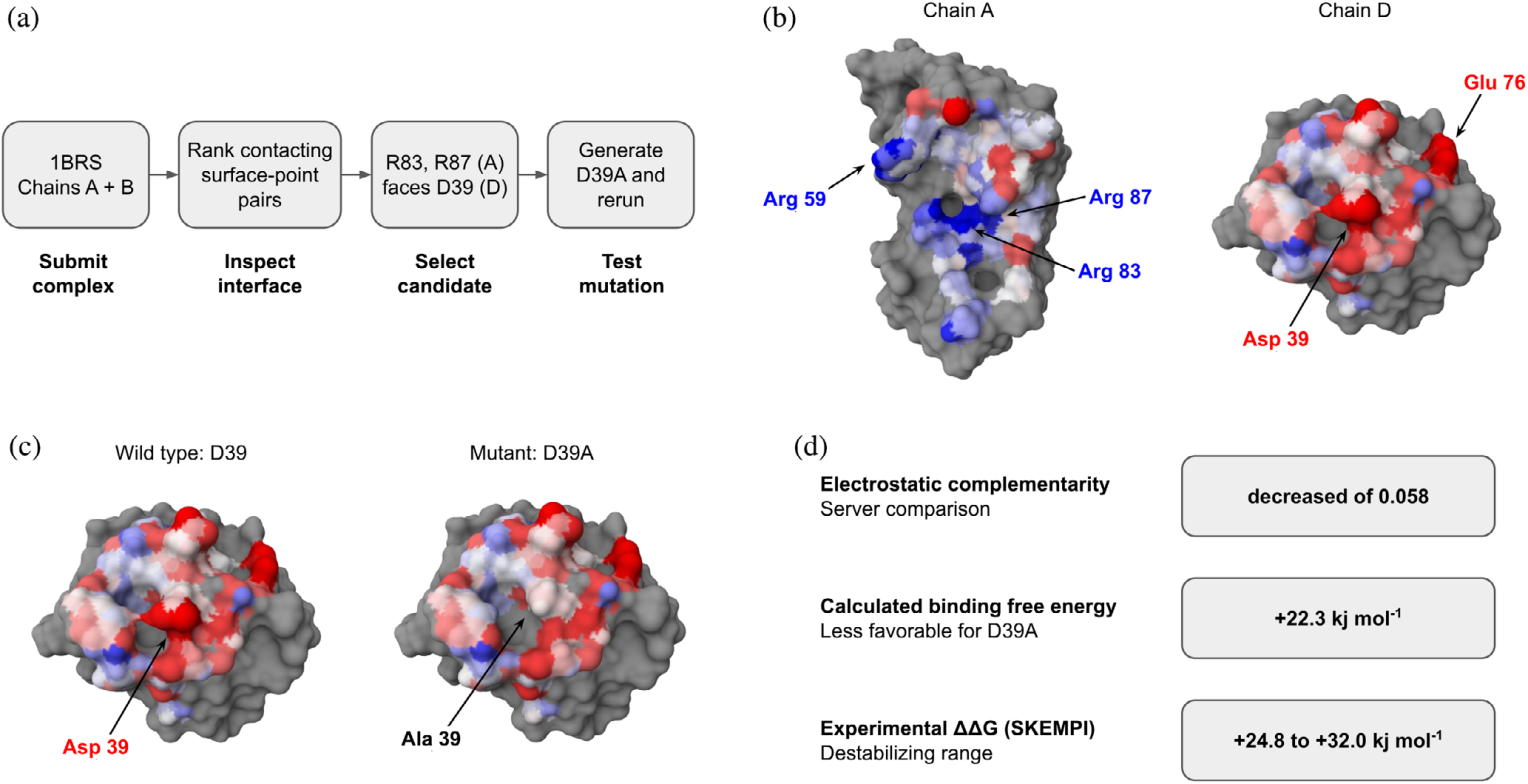
Bluues_cplx‐guided identification of a destabilizing Barnase/Barstar mutation. (a) Analysis workflow for the 1BRS complex formed by chains A and D, from interface characterization to selection and evaluation of the D39A substitution. (b) Open‐book representation of the wild‐type interface, showing the negative surface‐potential patch around D39 on chain D facing positive patches around R83 and R87 on chain A. (c) Matched wild‐type and D39A surface views generated using identical orientation, showing the reduction of the negative patch around residue 39. (d) Quantitative summary of the mutation effect. D39A decreases electrostatic complementarity by 0.058 and makes the calculated binding free energy 22.3 kJ/mol less favorable. SKEMPI reports an experimental destabilization of 24.8‐32.0 kJ/mol. Calculated and experimental values are presented as separate descriptors rather than as a quantitatively calibrated prediction.

The reference structure is PDB 1BRS, which contains three crystallographic complexes; chains A and D form the heterodimer analyzed here. On the Bluues_cplx input page, the user enters 1BRS in the PDB ID field and selects chains A and D as the two partners. The output reports several interface descriptors, including strong electrostatic complementarity at the interface, defined as the sign‐reversed Pearson correlation between electrostatic potentials at contacting surface points (0.646). These results suggest that electrostatics contributes substantially to complex formation and that reducing this complementarity may destabilize the complex. Sorting the pairs of contacting surface points by interaction score, defined as the negative product of their potentials, identifies the largest contributions from pairs involving E76 on chain D and R59 on chain A, and D39 on chain D and R87 or R83 on chain A.

The molecular viewer makes this pattern evident: D39 and E76 on chain D take part in a strongly negative potential patch at the interface, whereas the contacting residues R83 and R87 on chain A form a continuous positive patch; R59 participates in the same positive surface potential region. It is remarkable that the most destabilized mutant in the SKEMPI database (Moal & Fernández‐Recio, 2012) is the double mutant D39A on chain D and R59A on chain A. D39 on chain D is involved in two of the most important contacts, and therefore we selected this as a candidate position for a destabilizing mutation. Using ChimeraX (Meng et al., 2023), we generated a D39A mutant on chain D. To make the native and mutant calculations fully comparable, PQR files for both structures were generated with the same ChimeraX protocol.

The native and mutant PQR files were then submitted to Bluues_cplx. Comparison of the two outputs shows that the negative Pearson correlation between contacting surface‐point potentials is reduced by 0.058 in the mutant. The total binding free energy is also less favorable for the mutant by 22.3 kJ/mol.

Visual inspection of the surface potential around residue 39 on chain D confirms that the negative potential patch is strongly reduced in the D39A mutant.

Finally, SKEMPI confirms that D39A on chain D is destabilizing. It is the most destabilizing mutation involving charged residues in the Barnase/Barstar complex, with an unfavorable change in binding free energy between 24.8 and 32.0 kJ/mol depending on the experimental technique (Ikura et al., 2004; Schreiber & Fersht, 1995).

As discussed above, the absolute change in binding free energy computed by Bluues_cplx is not expected to be quantitatively accurate; using a different dielectric constant would give a different value. Nevertheless, the server output correctly identifies D39A as a highly destabilizing mutation: it is the second most destabilizing single‐point mutant among the 37 Barnase/Barstar entries available in SKEMPI (Moal & Fernández‐Recio, 2012). This retrospective example demonstrates the integration of residue prioritization, surface visualization, and rapid mutant comparison in a single workflow, rather than providing a new mechanistic interpretation of this well‐studied system.

## 3 CONCLUSIONS

Bluues2 provides the protein science community with a powerful and accessible platform for electrostatic analysis, combining the accuracy of surface integral Generalized Born methods with comprehensive functionality for single proteins and molecular complexes. The server addresses important needs by offering a practical alternative to full Poisson‐Boltzmann calculations that retains high accuracy while providing substantial computational speedups. This is particularly valuable given the explosion in available protein structures from both experimental determination and computational prediction, enabling rapid characterization that would be impractical with slower methods.

The extension to protein complexes fills a significant gap in currently available web tools. Few servers provide integrated analysis of intermolecular electrostatic interactions with quantitative metrics for electrostatic complementarity and binding free energy decomposition. These capabilities are particularly valuable for understanding molecular recognition, interpreting mutagenesis experiments, and guiding protein engineering efforts. The quantification of electrostatic complementarity through correlation of surface potentials provides insights that complement structural information and binding affinity measurements.

The organization and presentation of results make sophisticated electrostatic analyses accessible to researchers who may not be experts in computational biophysics. The combination of tabular summaries, sortable lists highlighting residues with particular properties, integrated three‐dimensional visualization, and comprehensive downloadable files means that both novices seeking quick overviews and experts wanting detailed quantitative data can efficiently extract needed information. Extensive documentation and worked examples further lower the barrier to entry.

Bluues2 provides a valuable tool for diverse applications. In structure‐function studies, it can identify electrostatic features contributing to biological activity. In protein engineering, rapid evaluation of electrostatic consequences of mutations can guide the design of variants with altered properties. Structure‐based drug design can benefit from the analysis of protein‐ligand electrostatics to optimize interactions contributing to affinity and selectivity.

The ongoing maintenance and development are supported by dedicated personnel and sustained funding, with integration into the DRMAAtic framework and shared technological components creating efficiency and enabling rapid deployment of improvements. As part of ELIXIR infrastructure, the server benefits from coordination with other European bioinformatics resources and adherence to standards for sustainability.

Bluues2 is freely available for non‐commercial use at https://bluues.biocomputingup.it/, with comprehensive documentation, example files, and source code for local installation.

## AUTHOR CONTRIBUTIONS

**Damiano Piovesan:** Conceptualization; methodology; software; visualization; writing – review and editing; writing – original draft; supervision. **Nima Rasekh:** Conceptualization; methodology; software. **Miguel Angel Soler:** Methodology. **Walter Rocchia:** Methodology. **Gennaro Esposito:** Methodology. **Federico Fogolari:** Methodology; writing – original draft; supervision. **Silvio C. E. Tosatto:** Funding acquisition; supervision.

## FUNDING INFORMATION

This study was supported by ELIXIR, the research infrastructure for life‐science data. European Union through NextGenerationEU, PNRR project ELIXIRxNextGenIT (IR0000010), and National Center for Gene Therapy and Drugs based on RNA Technology (CN00000041). Italian Ministry of Education and Research through the NextGenerationEU fund PRIN 2022 project: PLANS (2022W93FTW). Views and opinions expressed are, however, those of the authors only and do not necessarily reflect those of the European Union or the European Research Executive Agency. Neither the European Union nor the granting authority can be held responsible for them.

## Supporting information


**DATA S1.** 1BRS.

**DATA S2.** 1BRS_D_D_39_A.

**DATA S3.** Validation_data_bluues2.

## Data Availability

The Bluues2 server is accessible at https://bluues.biocomputingup.it/. Comprehensive documentation, example files, and tutorials are provided at the same URL. The source code for the underlying programs (bluues2 and bluues_cplx) is available for non‐commercial use and can be obtained for local installation from the server website as well as from GitHub URLs: https://github.com/federico-fogolari/bluues2 and https://github.com/federico-fogolari/bluues_cplx.
